# Impact of primary, specialist, and hospital care data on disease frequency estimates in older adults in Sweden

**DOI:** 10.1038/s41598-025-19621-3

**Published:** 2025-09-17

**Authors:** Katharina Schmidt-Mende, Maria Feychting, Eric Chen, Javier Louro, Karin Modig

**Affiliations:** 1 Academic Primary Health Care Centre, Stockholm Region, Stockholm, Sweden; 2 Division of Family Medicine and Primary Care, Department of Neurobiology, Care Sciences and Society, Karolinska Institutet, Huddinge, Sweden; 3 Unit of Epidemiology, Institute of Environmental Medicine, Karolinska Institutet, Stockholm, Sweden; 4 Institute of Environmental Medicine, Karolinska Institutet, Box 210, Stockholm, 17177 Sweden

**Keywords:** Diseases, Health care, Medical research, Risk factors

## Abstract

Nordic countries have a long-standing tradition of using administrative healthcare data from specialist and hospital settings to study disease frequency and etiological associations. While similar data are increasingly available elsewhere, large-scale primary care data remain limited. Understanding how data source affects disease identification is therefore critical. We conducted a register-based study including all individuals aged ≥ 60 years residing in Region Stockholm, Sweden, from 2017 to 2022. ICD-10 codes from primary care, specialist outpatient, and hospital care were grouped into 60 disease categories. One-year cumulative incidence and prevalence were estimated and compared. Disease patterns were broadly similar across care settings, with hypertension being the most common diagnosis. However, underestimation was generally greater for incidence than for prevalence. Hospital and specialist care data primarily captured acute and severe conditions, e.g., stroke, ischemic heart disease, falls, and cancer, while primary care data more effectively identified chronic risk factors such as diabetes, hypertension, hyperlipidemia, and psychiatric disorders, particularly for incident cases. Age-related differences in underestimation varied by disease but showed no consistent pattern. Primary, specialist, and hospital care data each capture distinct aspects of the disease landscape in older adults. Excluding primary care data leads to systematic underestimation of many common and chronic conditions, especially for newly diagnosed cases. This study provides guidance for epidemiological research using administrative health registers and highlights the importance of integrating data from multiple levels of care to improve the accuracy of disease burden estimates in epidemiological research using administrative health registers.

**Supplementary Information:**

The online version contains supplementary material available at 10.1038/s41598-025-19621-3.

## Introduction

Administrative health care data are widely used to describe disease frequency, and to investigate the etiological relationships between risk factors and disease outcomes. Sweden, together with the other Nordic countries, has a longstanding tradition of utilizing nationwide health data registers^1,2^. In recent years, however, an increasing number of countries have gained access to similar data sources including primary care data (for example Canada^3,4^, UK^5^ and Switzerland^6^. The significance of administrative health care data has grown, particularly in response to declining participation rates in surveys and other research cohorts. Moreover, administrative health registers allow researchers to study entire populations without the selection biases, thereby providing insights in real-world settings^7,8^. This is especially relevant as populations age, given that old individuals with multiple long-term conditions are underrepresented in both randomized controlled trials and research cohorts^9^.

Hospitalization data are perhaps the most used administrative health data^10,11^, sometimes supplemented by data on outpatient specialist care. Together, these sources form the foundation of the Swedish National Patient Register. Unfortunately, Sweden and other Nordic countries lack a national register for primary care. Efforts are underway to develop such a register, but to date, only regional primary care databases are available. The absence of comprehensive primary care data may result in underestimation of disease prevalence and incidence for conditions predominantly managed in primary care^12^, such as hypertension^13,14^ and mental disorders^15^. Understanding the magnitude of such underestimation is important^16^. Additionally, the degree of underestimation may differ for newly diagnosed cases vs. prevalent cases, and across population subgroups, particularly by age. Many conditions are first detected in primary care, while others are identified during hospitalization or specialist visits. Incidence estimates based only on secondary care may therefore underestimate diseases typically diagnosed in primary care, while conditions that commonly result in hospitalization may be more accurately captured.

Achieving as comprehensive a view as possible of disease prevalence in the population is crucial for obtaining a holistic understanding of overall health status. On the one hand, the likelihood of identifying older and frailer individuals and capturing their diagnoses may be higher than for younger individuals as they are hospitalized more frequently. On the other hand, these same individuals are often treated more extensively in primary care^17^, where their established relationships with providers may increase the likelihood of diagnosis in that setting.

In this study, we estimate and compare the incidence and prevalence of a range of diseases in adults 60 years and older using administrative health care data from hospitals, specialist outpatient clinics, as well as from primary care. The overall aim is to enhance the understanding of how the source of disease information affects the identification of diseases in administrative health records and for which diseases and ages discrepancies may be especially large. Specifically, we estimate and compare the 1-year cumulative incidence and prevalence of various diseases in the population aged 60 years and older in Region Stockholm, Sweden.

## Methods

### Study population

The entire population aged 60 years or older in the region Stockholm was identified in the register of the total population^18^. We calculated the distribution of any physical consultation with a physician according to the main ICD-chapters and level of care contact. For the prevalence estimations all individuals alive on 31 st of December 2022 (last available date) consisted of the study population (*n* = 497,736), and for incidence the population alive on January 1 st 2022 (*n* = 516,077), of whom 54% were women [current registered sex], with a mean age of 72 years and with 20% being 80 years and older.

### Health care data registers

Through the unique personal identification number assigned to all individuals in Sweden^19^, health care information was linked from two registers. 1) The National Patient Register (NPR) contains data on all hospital- (nationwide since 1987) and outpatient specialist care data (since 2001). With a few exceptions, diagnoses in the NPR have a positive predictive value ranging from 85 to 95%, indicating high validity^11^. 2) The Stockholm regional healthcare data warehouse (VAL) for primary care data. VAL includes data on all visits in primary care. All healthcare contacts are registered in the database, except for a few private healthcare providers without tax funding, some basic municipal home healthcare, and some healthcare given within special housing^20^. In both registers, diseases are classified according to the International Classification of Diseases (ICD-10). For each visit a main diagnosis is coded together with the possibility to up to 30 secondary diagnoses (although the majority of records contain no more than 4 secondary diagnoses).

### Diseases

Diseases were identified using registered visits and corresponding ICD-codes according to a definition developed by an expert team of geriatricians and general practitioners which categorize 918 ICD-codes into 60 groups of chronic diseases^21^. A detailed list of ICD-codes for each disease group are found in supplementary Table 1.

### Analyses

#### Distribution of care records according to ICD-codes and care level

We explored how all health care records during 2022, in each care level (hospital, specialist outpatient, primary care) were distributed according to ICD-chapters of disease groups. This analysis was conducted using both the main diagnostic code alone and considering secondary diagnostic codes. All ICD-codes except O00–O99, related to pregnancy and childbirth, and P00–P96, related to conditions originating in the perinatal period, were considered.

#### Cumulative incidence of diseases

For each of the 60 disease groups, we estimated 1-year cumulative incidence during the year 2022 as the ratio between all newly registered diagnostic codes belonging to the disease group in 2022 to the total population alive on January 1 st, 2022. A diagnosis was considered new if the person had not had any of the diagnostic codes belonging to the disease group recorded at any time between January 1 st, 2017 until December 31 st, 2021. Both main and secondary diagnoses were considered when identifying the disease.

### Prevalence

The prevalence of each disease group in the population was calculated as the ratio of individuals recorded as having any of the diagnostic codes belonging to the disease group between January 1st 2017 until December 31 st 2022 to the total population as of December 31 st 2022. Comparing the results of the prevalence analysis to the incidence aimed to assess the effect of time on the likelihood of identifying diseases, since many diseases are initially diagnosed in primary care, and eventually referred to specialist care. Both main and secondary diagnoses were considered when identifying the disease.

Both cumulative incidence and prevalence were estimated based on disease groups identified in the following data sources:

i] NPR: hospital and specialist outpatient care only^11^.
ii] VAL: primary care only.
iii] NPR $\:\cap\:$ VAL: Both of the sources.
iv] NPR $\:\cup\:$ VAL: Either of the sources.

### Underestimation of incidence and prevalence without primary care

To compare the discrepancy for cumulative incidence and prevalence respectively, depending on the source of information, we calculated, for each disease group, the proportion that was missed without having access to primary care data using the following formula:

$$\begin{aligned} {\text{Proportion~of~}}1 & - {\text{year~cumulative~incidence~lost~without~primary~care}} \\ & = 1 - \frac{{{\text{Cumulative~incidence~through~NPR~}}}}{{{\text{Cumulative~incidence~through~NPR}} \cup {\text{VAL}}}} \\ \end{aligned}$$

$${\text{Proportion~of~prevalence~lost~without~primary~care=1-}}\frac{{{\text{Prevalence~through~NPR~}}}}{{{\text{Prevalence~through~NPR}} \cup {\text{VAL}}}}$$

We assumed the true level to be that consisting of all available care records. Based on this, we estimated the deviation that occurs when using only hospital and outpatient specialist care data. Additionally, we stratified individuals by age—those below and above 80 years—since primary care utilization may vary with age. The 80-year age cut-off is partly arbitrary but reflects a commonly used distinction in research between “younger old” (65–79) and “older old” (80+), where care needs and treatment approaches often differ.

The study was performed in line with the principles of the Declaration of Helsinki. Approval was granted by the Regional Ethics Committee of Stockholm No 2011136−31/5, and supplement by the National Ethics Authority Dnr 2022-03486-02. The need to obtain informed consent was waived by the Regional Ethics Committee of Stockholm and the National Ethics Authority.

## Results

**Fig. 1:**
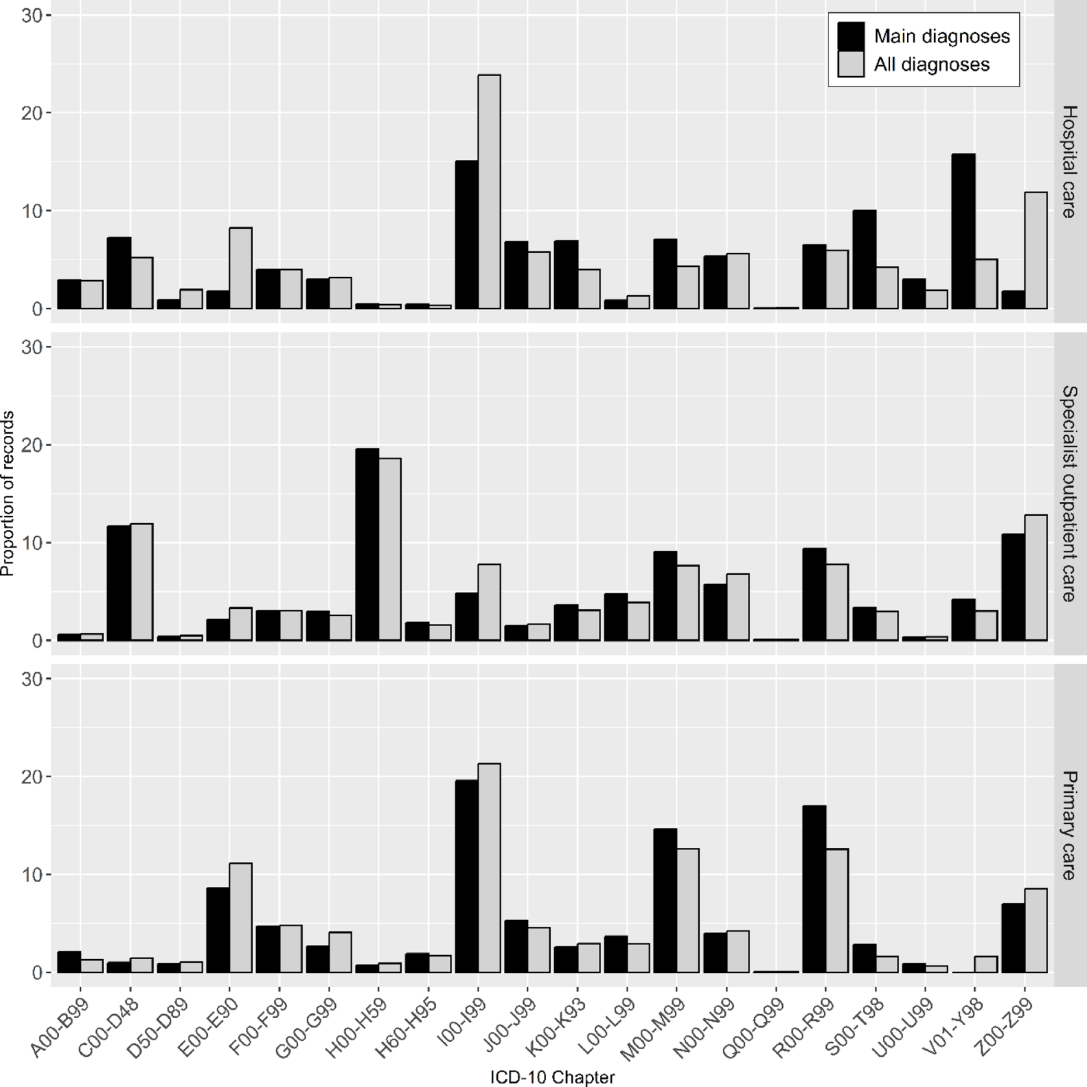
Proportion of all records by ICD-chapters in hospital, specialist outpatient, and primary care respectively, for main diagnoses only, and when considering also secondary diagnoses for the population 60 years and older in region Stockholm, Sweden. A00–B99: Certain infectious and parasitic diseases, C00–D48: Neoplasms, D50–D89: Diseases of the blood and blood-forming organs, E00–E90: Endocrine, nutritional and metabolic diseases, F00–F99: Mental and behavioural disorders, G00–G99: Diseases of the nervous system, H00–H59: Diseases of the eye and adnexa, H60–H95: Diseases of the ear and mastoid process, I00–I99: Diseases of the circulatory system, J00–J99: Diseases of the respiratory system, K00–K93: Diseases of the digestive system, L00–L99: Diseases of the skin and subcutaneous tissue, M00–M99: Diseases of the musculoskeletal system, N00–N99: Diseases of the genitourinary system, Q00–Q99: Congenital malformations, deformations, R00–R99: Symptoms, signs and abnormal clinical findings, S00–T98: Injury, poisoning and external causes, V01–Y98: External causes of morbidity and mortality,, Z00–Z99: Factors influencing health and contact with health services, U00–U99: Codes for special purposes.

Figure 1 displays the distribution of total care records according to ICD-chapter for the different care levels, for main diagnoses alone, and when considering also secondary diagnoses. In hospitalizations, cardiovascular diseases (I-chapter), followed by falls (W), constitute most of the recorded diagnoses when *main* ICD codes are considered; 15% and 11%, respectively. When considering both *main and secondary* diagnoses, cardiovascular disease (I) constitutes an even larger proportion (24%), while falls (W) decrease to 3%. Instead, factors influencing health status and contact with health services (Z-chapter) and endocrinological disease (E) have a larger share of all hospital records, 12% and 8% respectively. In specialist outpatient care, ear and eye conditions (H) followed by factors influencing health status and contact with health services (Z) account for most of the records when *main* diagnoses are considered, 21% and 11% respectively. No major changes in patterns occur when including *secondary* diagnoses. In primary care, cardiovascular diseases (I) account for most of the visits (20%), followed by unspecific symptom diagnoses (R) (17%) when *main* diagnoses are considered. No major changes in patterns occur when also considering *secondary* diagnoses.

Analyzing general differences between hospital and primary care reveals that several chapters represented a small proportion of coded records in primary care data, including certain infectious and parasitic diseases (A, B), neoplasms (C, D), injuries (S, T), codes for special purposes (U) and external causes of morbidity and mortality (V-Y). Contrary, bone, joint, or muscle disorders (M) and endocrinological disease (E) are mainly handled in primary care, with few registrations in hospital care.

**Fig. 2:**
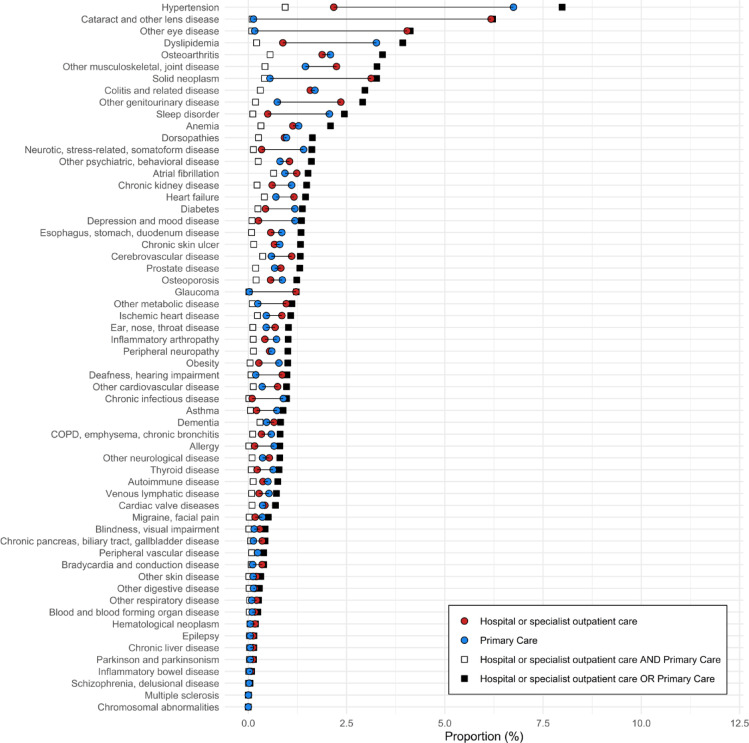
1-year cumulative incidence of diseases depending on source of information for the population 60 years and older in region Stockholm, Sweden in the year 2022.

In Fig. 2, hospital care and specialist outpatient care are combined. The red dot indicates the cumulative incidence of diseases when hospital or specialist outpatient care is the data source, while the blue dot represents estimates based on primary care data only. The black square shows the cumulative incidence when all data sources are combined, representing the closest approximation to the true cumulative incidence obtainable from administrative health registers. The white square shows the cumulative incidence when requiring the case to be recorded in both specialist care and primary care within the same year. The highest cumulative incidence, when combining sources, is observed for hypertension, 8.0%, followed by cataract, 6.2% and other eye diseases 4.1%. For hypertension specifically, the cumulative incidence is 2.2% when identified through hospital or specialist outpatient care alone, and 6.2% when identified through primary care alone. The share of patients identified in both sources is very low, indicating that there are different patients identified in the respective care setting, and that during one calendar year individuals are unlikely to be recorded in both settings for the same diagnosis. Worth noting is that for some diseases the cumulative incidence is higher when primary care is the source, for example hypertension and dyslipidemia, and psychiatric disease such as somatoform disease as well as sleep disorders. For other diseases the cumulative incidence is higher when hospital or specialist outpatient care is the source, for example cataract, neoplasms, and other musculoskeletal/joint disease. Details underlying the figure can be found in supplementary Table 2.

**Fig. 3:**
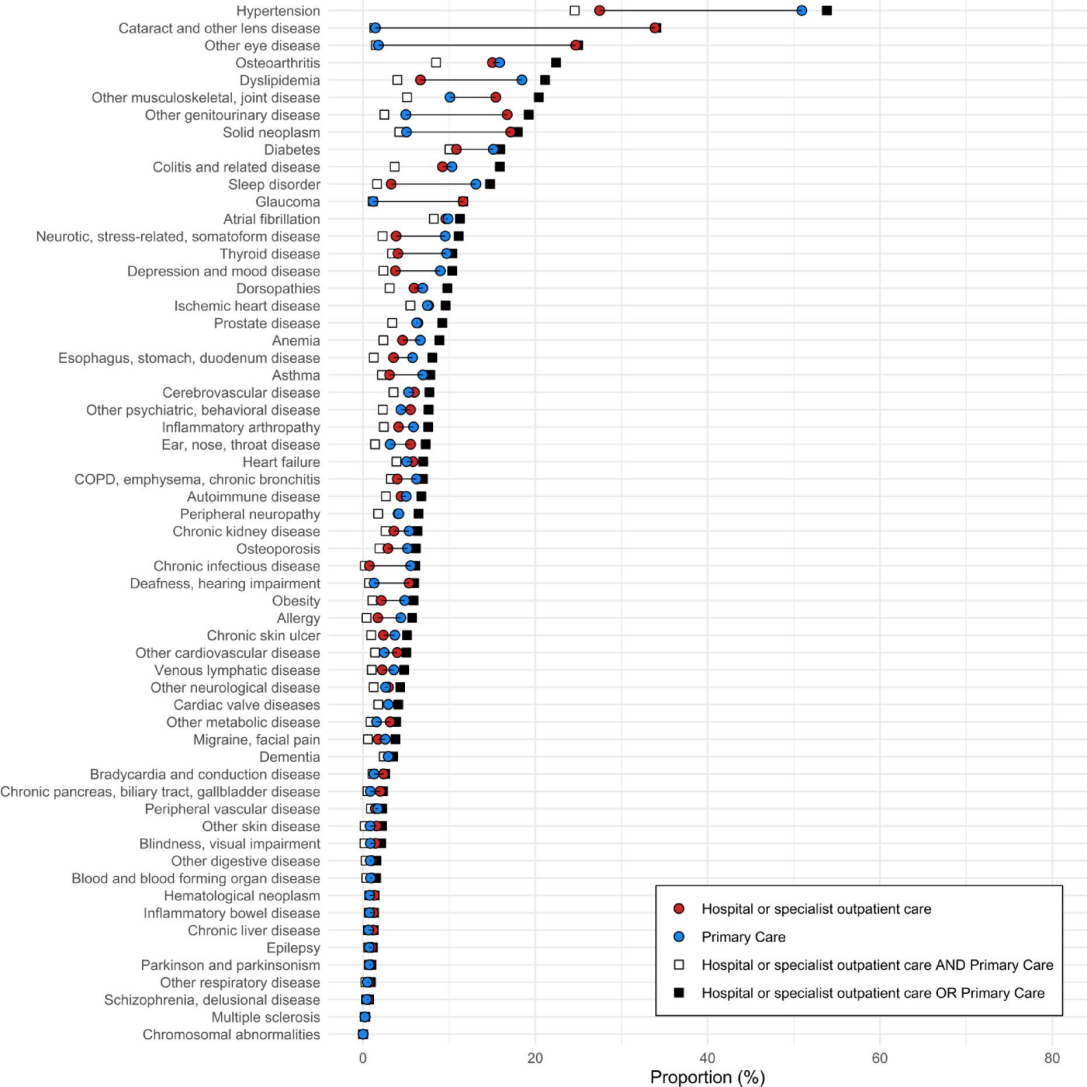
Prevalence of diseases, depending on source of information, in the population 60 years and older in region Stockholm, Sweden December 31, 2022. Diagnoses recorded during the years 2017–2022.

Similarly, Fig. 3 shows the prevalence of each one of the diseases of interest. The top list is similar to Fig. 2, meaning that the caregiver diagnosing the patient is the same regardless of whether it is an incident or prevalent disease, albeit at higher levels in Fig. 3 which represents a longer diagnostic period. However, for some diseases the pattern differs. Severe cardiovascular diseases, e.g. ischemic heart disease, are more often diagnosed first in hospital or specialist outpatient care. Of note, the share of cases identified in both care settings [white square] is higher for prevalent cases (Fig. 3) than for incident cases (Fig. 2) reflecting that beyond the one-year period more people have a record in both primary care and hospital/outpatient specialist care. The most prevalent condition is hypertension, with an overall prevalence of 53.8%. The prevalence using primary care data is 50.9%, whereas the prevalence using hospital or outpatient specialist care data is 27.5%. Details of the figure can be found in supplementary Table 3.

**Fig. 4:**
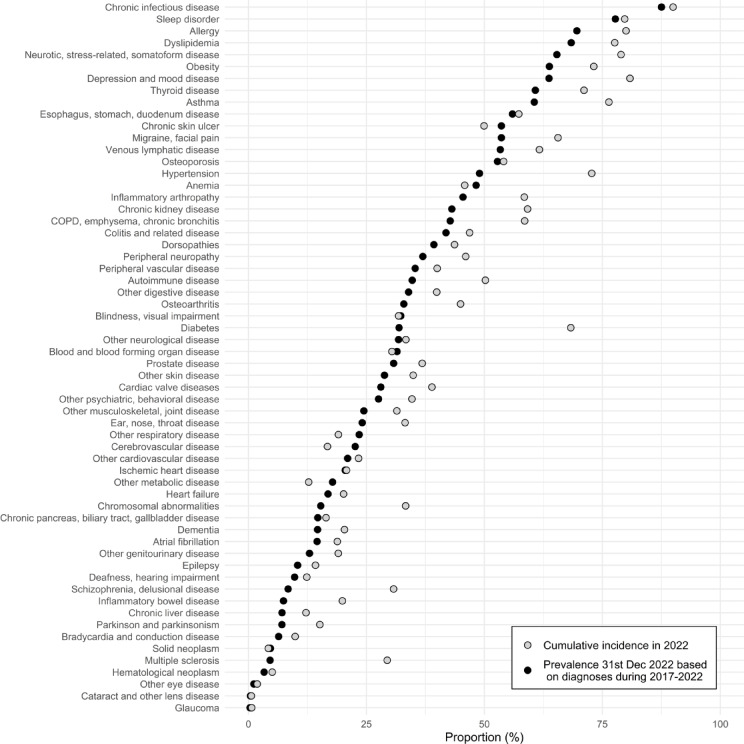
Proportion of cumulative incidence [grey] and prevalence [black] for various disease groups that is missed in the absence of primary care data, for the population 60 years and older in region Stockholm, Sweden in the year 2022.

Figure 4 shows the degree of underestimation (in a relative way) in cumulative incidence (grey) and prevalence (black) when primary care data are excluded. For 19 of the 60 disease groups more than 50% of cumulative incidence is missed without primary care data (for detailed numbers underlying the Figure see Supplementary Table 2, column “1-(PR/OR)”). For prevalence the corresponding number is 14 out of 60 diseases (for detailed numbers underlying the Figure see Supplementary Table 3, column “1-(PR/OR)”). In general, underestimation is greater for incidence than for prevalence across most diseases, although the magnitude of this difference varies considerably. The greatest loss in both incidence and prevalence is observed for chronic infectious diseases, where 90% and 88%, respectively, go undetected when primary care data are excluded. This is largely attributable to Lyme disease—specifically its early manifestation, erythema migrans—which accounts for 80% of the total prevalence in this disease category. Substantial underestimation is also seen for psychiatric disorders, cardiovascular risk factors, and allergic conditions. For example, omitting primary care data leads to underestimation of cumulative incidence and prevalence by 81% and 64% for depression, 80% and 70% for allergies, 78% and 68% for dyslipidemia, and 80% and 78% for sleep disorders. In contrast, the exclusion of primary care data has minimal impact on estimates for neoplasms and eye diseases, with incidence/prevalence losses of only 4%/5% and 2%/1%, respectively. For some conditions, such as sleep disorders and osteoporosis, the degree of underestimation is similar for incidence and prevalence. However, for others—such as diabetes and hypertension—the underestimation is substantially greater for incidence than for prevalence.

**Fig. 5:**
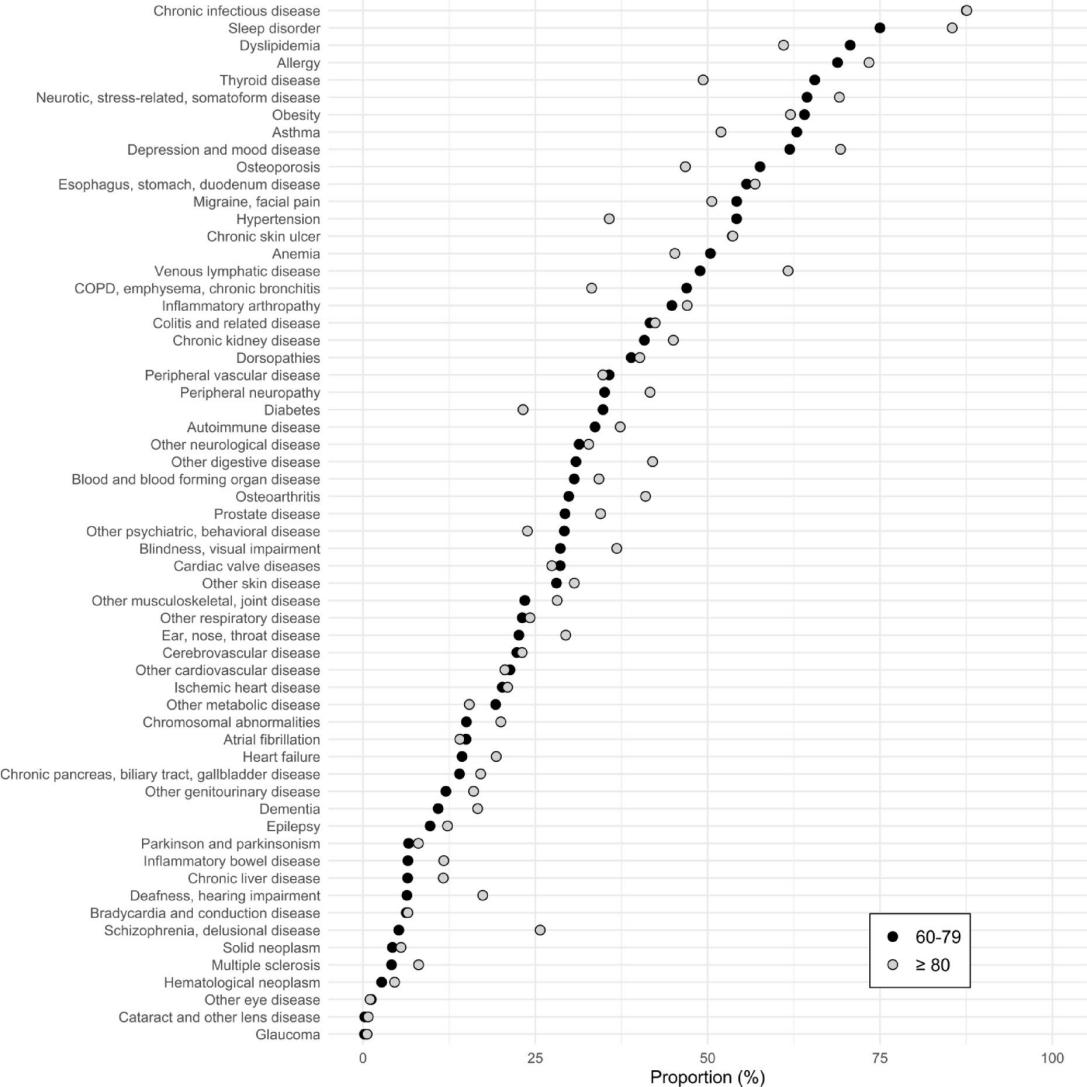
Proportion of prevalence for various disease groups that is missed in the absence of primary care data stratified by age groups 60–79 [black] and 80 and older [grey], region Stockholm, Sweden in the year 2022.

Figure 5 illustrates the underestimation of disease prevalence—corresponding to the black squares in Fig. 4—in the absence of primary care data, stratified by age groups 60–79 years and ≥ 80 years. For certain disease groups, such as chronic infectious diseases, the degree of underestimation is similar across both age groups. However, notable differences emerge for other conditions. In some cases, underestimation is greater among the oldest individuals [≥ 80 years], as seen for sleep disorders, schizophrenia, and venous or lymphatic diseases. In contrast, conditions such as thyroid disorders, hypertension, chronic obstructive pulmonary disease (COPD), and diabetes exhibit greater underestimation in the 60–79-year age group. Notably, for severe cardiovascular diseases—such as cerebrovascular disease—the difference in underestimation between age groups is minimal.

## Discussion

This study aimed to enhance the understanding of how the source of disease information – hospital or specialist outpatient care versus primary care – affects the identification of patients with various diseases in administrative health registers. While the overall disease patterns captured by the two sources were similar for both incident and prevalent cases, with hypertension being the most common diagnosis, each data source reflects different aspects of the disease landscape over time. Hospital and specialist outpatient care data primarily capture acute and severe events, such as stroke, ischemic heart disease, falls, and cancer. In contrast, primary care data more effectively capture underlying risk factors, including diabetes, hypertension, hyperlipidemia, and psychiatric diseases. An exception to this is eye diseases, particularly conditions such as cataract, which is most often handled within specialist outpatient care. For many diseases, the absence of primary care data results in a greater underestimation of incidence than prevalence. For example, approximately 70% of new cases of hypertension and diabetes are missed when primary care data are excluded, whereas “only” 48% (hypertension) and 32% (diabetes) are missed for prevalent cases. This suggests that primary care often serves as the initial point of diagnosis. Over time, these cases may eventually be identified in hospital or specialist outpatient settings, especially as the conditions progress or require more specialist care.

It is well established that disease frequency in the population is underestimated when primary care data are unavailable^15^. This limitation is problematic when assessing disease burden, both in terms of the total burden each disease imposes and the specific burden certain conditions place on primary care services. For instance, psychiatric diseases are highly prevalent and resource-intensive in primary care, yet they are often underrepresented in prevalence estimates, which are frequently based solely on specialist care data^22^. For some diseases the overlap of patients identified both in hospital or specialist outpatient care AND in primary care is low indicating that different patients are identified in the respective data source. While our study could not determine whether these discrepancies reflect systematic differences based on sociodemographic factors such as sex or education level, it is plausible that such patterns exist and would influence patient characterization if only one data source is used. From an etiological research perspective, underestimation of case numbers may not necessarily bias results if all subgroups are adequately represented and statistical methods can adjust for potential differences. In this study, we investigated age as a potential source of systematic bias. Psychiatric diseases were more likely to be underestimated in individuals aged 80 years and older compared to those aged 60–79 years when primary care data were excluded. Beyond this, no consistent age-related patterns of underestimation were observed. This may reflect current clinical guidelines on multimorbidity^23^, which emphasize individualized treatment approaches rather than age-based decision-making. However, psychiatric comorbidity plays a critical role in recovery following severe somatic conditions. Therefore, the inability to reliably assess psychiatric diagnoses—such as depression following myocardial infarction or stroke—may result in biased estimates of disease prognosis and affect our understanding of long-term outcomes^24^.

It is difficult to determine how generalizable our findings are to other regions or countries. While the overall pattern—where certain diseases are more frequently captured in primary care and therefore underestimated when relying solely on hospital or specialist outpatient data—is likely similar across healthcare systems, the magnitude of underestimation is expected to vary. A study published 2007 from another Swedish region compared the prevalence of four chronic diseases when using primary care data versus hospital/outpatient specialist care^14^. The underestimation of the prevalence in the absence of primary care data was broadly consistent with findings. For example, the reported underestimation for type 2 diabetes was 23% in the earlier study compared to 31% in ours; for hypertension, 68% versus 49%; for asthma, 53% versus 61%; and for chronic obstructive pulmonary disease , 48% versus 43%. These differences are likely attributable to variations in the study populations—specifically, the inclusion of all ages in the 2007 study versus individuals aged 60 years and older in our analysis—as well as changes in diagnostic practices over time. The degree of underestimation of disease prevalence is likely to vary across countries depending on healthcare system, particularly the strength of primary care “gatekeeping“^25^. By gatekeeping, we refer to whether access to outpatient specialist care typically requires a referral from primary care. During the study period, referrals were generally required for outpatient specialist care, with only a few exceptions^26^. However, patients presenting directly to emergency departments may bypass primary care. In healthcare systems with strong gatekeeping mechanisms, the disparity in disease identification between primary care and specialist care is likely to be more pronounced. Conversely, in systems where patients have direct access to specialists, these differences may be less marked. Moreover, healthcare systems that offer more extensive health screening within primary care may report higher disease prevalence due to earlier and more systematic detection^27^. In Sweden, there are no general health screening programs conducted through primary care; rather, patients are typically assessed after seeking care for specific symptoms^28^. Finally, our data are drawn from a single healthcare region—a metropolitan area with high accessibility to specialist care. This context may underestimate the diagnostic role of primary care compared to regions where specialist care is more limited, and primary care therefore serves a more central diagnostic function, one example is the role of memory clinics versus primary care for dementia diagnosis where more rural areas in Sweden have less diagnoses of dementia identified in specialist outpatient clinics^29^.

However, the purpose of our study was not to provide exact estimates of incidence and prevalence, but to demonstrate for which diseases there is a discrepancy between identification in primary versus specialist care. This overall pattern, we believe, could be generalized to some extent, but only with caution, taking into account factors such as accessibility of care, the presence of specialist clinics, and reimbursement systems.

### Strengths and limitations

Although the quality of the data underlying this study is high, limitations are always present. In addition to the above discussed concerns regarding generalizability, coding practices should be considered when interpreting or generalizing the results.

Coding practices for ICD diagnoses vary across healthcare settings, which may influence the completeness and comparability of administrative data. In primary care, diagnoses are often limited to a single, main diagnosis due to time constraints during typically brief consultations that focus on the primary reason for the visit. As a result, the full clinical picture, including comorbidities, may not be consistently recorded. Conversely, financial incentives can encourage the inclusion of multiple diagnoses, as reimbursement may increase with the number of coded comorbidities. In specialist care, diagnostic coding tends to reflect the clinician’s area of expertise. For example, cardiologists may be more likely to include cardiovascular-related comorbidities such as renal failure. Additionally, specialist and emergency care settings tend to prioritize the identification and coding of acute and severe conditions, which may lead to underreporting of milder or chronic comorbidities. At the same time, in hospital settings, administrative staff may review diagnoses recorded by healthcare providers, compare them with clinical notes, and add codes retrospectively, to ensure appropriate reimbursement. In contrast, primary care settings typically lack such administrative oversight, further impacting coding consistency. Moreover, primary care frequently relies on symptom-based diagnoses [R-chapter codes], when further investigations are needed to assign a definite diagnosis. Retrospective updates of symptom diagnoses are rarely carried out in practice, resulting in an underestimation of disease measures. Examining individuals with symptom-based diagnoses may enhance our understanding of disease patterns.

Furthermore, the overall quality of ICD coding, particularly in primary care, is variable and differs across diagnoses. Complementary sources such as the Prescribed Drug Register, disease-specific quality registers, and population surveys may improve case ascertainment and help validate register-based estimates. In addition, the use of different electronic health record systems across Swedish regions introduces heterogeneity that complicates comparisons. At the same time, Swedish register data also have notable strengths. Unlike in many other countries, coded information is available not only from inpatient care but also from outpatient specialist care, providing a more comprehensive coverage of diagnoses. However, the absence of a national primary care register remains a major limitation, and regional differences in availability and coding practices further restrict comparability. Taken together, these strengths and limitations underline the importance of cautious interpretation and the need for harmonization of data sources. Future work could usefully compare patterns of concordance across countries to clarify how healthcare organization and data infrastructures shape disease estimates.

## Conclusion and clinical implications

This study demonstrates that the inclusion of primary care data is essential for accurately estimating the frequency of many common diseases in older adults. While patterns of diagnosis across care settings are generally complementary, each setting captures distinct segments of the disease landscape. Primary care data are particularly important for identifying chronic and less acute conditions, including cardiovascular risk factors, psychiatric disorders, allergic conditions, and early-stage infections such as Lyme disease. In contrast, hospital and specialist outpatient care primarily capture acute and severe conditions such as neoplasms and cardiovascular events. Our findings show that excluding primary care data may result in substantial underestimation of both cumulative incidence and prevalence for a broad range of conditions—particularly for incidence, and especially among adults aged 60–79 for certain chronic diseases. Moreover, the overlap between patients identified in primary versus specialist care is limited, highlighting that relying on a single data source can lead to incomplete and potentially biased estimates. These results underscore the need for integrating multiple levels of care data in register-based research to obtain a more complete and accurate picture of disease burden and patterns across the population.

## Supplementary Information

Below is the link to the electronic supplementary material.

Supplementary Material 1

## Data Availability

The individual level data underlying this study cannot be shared publicly because of the General Data Protection Act in Sweden. Access to the data can be permitted to external researchers after ethical vetting and establishment of a collaboration agreement. Contact the corresponding author for questions about data sharing or sharing of data code.
